# Adiponectin reduces apoptosis of diabetic cardiomyocytes by regulating miR-711/TLR4 axis

**DOI:** 10.1186/s13098-022-00904-y

**Published:** 2022-09-16

**Authors:** Yu Zuo, Tao Xiao, Xiangdong Qiu, Zuoliang Liu, Shengnan Zhang, Na Zhou

**Affiliations:** 1 Department of the Pre-Hospital First-Aid, The Third Xiangya Hospital of Central South University, Changsha, Hunan 410013 People’s Republic of China; 2 Nursing Department, The Third Xiangya Hospital of Central South University, No. 138, Tongzipo Road, Yuelu District, Changsha, Hunan 410013 People’s Republic of China; 3 Intensive Care Unit, The Third Xiangya Hospital of Central South University, Changsha, Hunan 410013 People’s Republic of China; 4 Department of Anesthesiology, Hunan Aerospace Hospital, Changsha, Hunan 410205 People’s Republic of China

**Keywords:** Adiponectin, miRNA-711, TLR4, Cardiomyocyte, Diabetes, Apoptosis

## Abstract

**Objective:**

To investigate the regulation of adiponectin/miR-711 on TLR4/NF-κB-mediated inflammatory response and diabetic cardiomyocyte apoptosis.

**Methods:**

Diabetes models were established using rats and H9c2 cardiomyocytes. qRT-PCR was used to detect adiponectin, miR-711, and TLR4. MTT, β-galactosidase staining, and flow cytometry were utilized to assess cell viability, senescence, and apoptosis, respectively. The colorimetric method was used to measure caspase-3 activity, DCFH-DA probes to detect ROS, and western blotting to determine the protein levels of Bax, Bcl-2, TLR4, and p-NF-κB p65. ELISA was performed to measure the levels of adiponectin, ICAM-1, MCP-1, and IL-1β. Dual-luciferase reporter system examined the targeting relationship between miR-711 and TLR4. H&E and TUNEL staining revealed myocardial structure and apoptosis, respectively.

**Results:**

Adiponectin and miR-711 were underexpressed and TLR4/NF-κB signaling pathway was activated in high glucose-treated H9c2 cells. High glucose treatment reduced viability, provoked inflammatory response, and accelerated senescence and apoptosis in H9c2 cells. miR-711 could bind TLR4 mRNA and inactivate TLR4/NF-κB signaling. Adiponectin treatment increased miR-711 expression and blocked TLR4/NF-κB signaling. Adiponectin/miR-711 reduced myocardial inflammation and apoptosis in diabetic rats.

**Conclusion:**

Adiponectin inhibits inflammation and alleviates high glucose-induced cardiomyocyte apoptosis by blocking TLR4/NF-κB signaling pathway through miR-711.

## Introduction

Diabetes mellitus (DM) embraces heterogeneous metabolic disorders characterized by chronic hyperglycemia [1]. DM is commonly classified into two types: type 1 DM (T1DM), caused by an absolute deficiency of insulin ensuing destruction of pancreatic beta cells, and type 2 DM (T2DM), resulting from insulin resistance and subsequent deficiency of insulin secretion [2]. T2DM accounts for 90% of all diabetic cases and presents a rising incidence, especially in Asia [3]. Individuals with diabetes are more likely to encounter cardiovascular diseases and microvascular complications which lead to increased mortality and decreased overall quality of life [4]. Many cellular mechanisms such as oxidative stress, inflammation, insulin signaling, autophagy, and myocardial substrate metabolism are dysregulated in models and clinical samples of diabetic cardiomyopathy [5]. These cellular processes can be targeted to avoid diabetic heart failure and improve the outcomes of diabetes.

Adiponectin is a 30-kDa monomeric glycoprotein secreted mainly by adipose tissue into the circulation where it is abundantly expressed [6]. Apart from adipocytes, adiponectin can also be produced by human and murine osteoblasts, skeletal and cardiac myocytes, and placental tissue [7]. Adiponectin maintains insulin sensitivity in muscle cells by regulating lipid metabolism through signal transduction, so a decline in its expression is frequently observed in T2DM and metabolic syndrome [8]. Adiponectin also defends the cardiovascular system by exerting anti-inflammatory, anti-apoptotic, and antioxidant effects [9]. Therefore, adiponectin can be used to prevent cardiovascular complications in T2DM, which provokes our interest in its regulatory mechanism.

MicroRNAs (miRNAs) are short RNAs of 19–25 nucleotides that can be guided to the 3’ end of target mRNAs under the principle of base-pairing in their mature state and induce mRNA destabilization or translational repression, thereby post-transcriptionally silencing genes involved in functional interacting pathways [10, 11]. MiRNAs such as miR-320, miR-30c, and miR-200c have been shown to regulate cardiac lipotoxicity and hypertrophy in diabetes [12–14]. A study by Boursereau and colleagues showed that knockout of adiponectin activated Toll-like receptor 4 (TLR4)/nuclear factor (NF)-κB signaling pathway and induced muscle inflammation by decreasing the expression of miR-711 [15], but little is known about whether miR-711 is regulated by adiponectin in diabetic cardiomyopathy.

This study was designed to determine the influence of adiponectin/miR-711/TLR4 dysregulation on inflammatory response and biological behaviors of diabetic cardiomyocytes. The findings may prepare the way for new therapeutic strategies to manage cardiovascular complications in diabetes.

## Materials and methods

### Cell source and culture

Rat H9c2 cardiomyocytes were acquired from Nanjing Cobioer Biosciences Co., Ltd. (Jiangsu, China) and cultured in DMEM containing 10% fetal bovine serum (FBS) at 37 °C with 5% CO_2_. The cells were assigned to control group (cultured in low glucose-DMEM), osmotic (OS) group (cultured in 30 mmol/L mannitol-DMEM), and high glucose (HG) group (cultured in 30 mmol/L glucose-DMEM).

### Cell transfection

H9c2 cells were seeded into a 6-well plate (3 × 10^5^ cells and 2 mL of complete DMEM per well) and grew to 70% ~ 80% confluence. Mimic-negative control (NC) (5′-TTAGAACTUAAGTGGCGACGGA-3′), miR-711 mimic (5′-GGGACCCUGGGAGAGAUGUAAG-3′), inhibitor-NC (5′-GAATGTAGAGAGGGTCCCAGGG-3′), or miR-711 inhibitor (5′-CUUACAUCUCUCCCAGGGUCCC-3′) (Sangon, Shanghai, China) was delivered into the cells using Lipofectamine 2000 (Thermo Fisher, USA) and the cells were collected after 48 h. Adiponectin (10 μg/mL; human-derived; MedChemExpress, Monmouth Junction, NJ, USA) was administered before transfection and the drug treatment lasted for 15 min.

### Quantitative real-time polymerase chain reaction (qRT-PCR)

TRIzol (Invitrogen, Carlsbad, CA, USA) was used for total RNA extraction. Qualified RNA samples were diluted and reverse transcribed using a reverse transcription kit (miScript PCR Starter Kit, QIAGEN). qRT-PCR was performed following the instructions of IQ SYBR Green Supermix kit (BIO-RAD, USA). The reaction consisted of pre-denaturation at 95 °C (3 min) and 40 cycles of denaturation at 95 °C (15 s), annealing at 60 °C (30 s), and extension at 72 °C (30 s). U6 was used as an internal reference for miR-711 and GAPDH [16–19] for the remaining mRNAs. The 2^−ΔΔCt^ method was adopted for data analysis (ΔΔCT = ΔCt _experimental group_—ΔCt _control group_, ΔCt = Ct _target gene_—Ct _reference gene_). The experiment was repeated thrice. See Table 1 for the primer sequences.

**Table 1:** Primer sequences

Name of primer	Sequences
Adiponectin-F	5ʹ-GCCCAGTCATGAAGGGATTA-3ʹ
Adiponectin-R	5ʹ-TCTCTCCAGGAGTGCCATCT-3ʹ
miR-711-F	5ʹ-CGGGACCCTGGGAGAGA-3ʹ
miR-711-R	5ʹ-GTCGTATCCAGTGCAGGGTCCGAGGTATTCGCACTGGATACGACCTTACA-3ʹ
U6-F	5ʹ-CTCGCTTCGGCAGCACA-3ʹ
U6-R	5ʹ-AACGCTTCACGAATTTGCGT-3ʹ
TLR4-F	5ʹ-GAGGACTGGGTGAGAAACGA-3ʹ
TLR4-R	5ʹ-GAAACTGCCATGTCTGAGCA-3ʹ
GAPDH-F	5ʹ-AGTCTACTGGCGTCTTCACC-3'
GAPDH-R	5ʹ-CCACGATGCCAAAGTTGTCA-3'

*F* forward primer, *R* reverse primer

### Western blotting

Tissues or cells were incubated with RIPA buffer containing PMSF on ice for 30 min and centrifuged at 8000 g, 4 °C for 10 min. Supernatant was taken for protein quantification with a BCA kit (Thermo Fisher, USA). Proteins (50 μg) were dissolved in 2 × SDS loading buffer and boiled at 100 °C for 5 min. After SDS-PAGE electrophoresis, the proteins were transferred onto a PVDF membrane in wet conditions. The membrane was placed in 5% skimmed milk at room temperature for 1 h and incubated with antibodies against TLR4 (1:1000, ab13867, Abcam, USA), phosphorylated (p)-NF-κB p65 (1:1000, ab76302, Abcam), Bax (1:1000, ab32503, Abcam), Bcl-2 (1:1000, ab196495, Abcam) and GAPDH (1:1000, ab8245, Abcam) at 4 °C overnight. After being washed, the membrane was incubated with horseradish peroxidase (HRP)-labeled IgG (H + L) at room temperature for 2 h. The membrane was treated with ECL reagent and scanned by a gel imaging system. The bands were quantified by the Image J software. The experiment was repeated thrice.

### MTT test of cell viability

Cells in the log phase were seeded into a 96-well plate (5 × 10^4^ cells/well) and incubated for 24 ~ 36 h. Then 50 μL of MTT solution was added into each well and reacted at 37 °C for 4 h. Supernatant was removed and 150 μL of DMSO was added into each well. The plate was shaken at a low speed for 10 min to dissolve the formazan crystals. Optical density at 570 nm of each well was measured by a microplate reader. The experiment was repeated thrice.

### Senescence detection

Cells were cultured in a 6-well plate for 48 h and washed with PBS. Then 1 mL of fixative was added into each well and the plate was kept at room temperature for 20 min. The cells were washed with PBS and incubated with 1 mL of staining solution (β-galactosidase kit, Beyotime, Shanghai, China) overnight in a non-CO_2_ incubator at 37 °C. The staining solution consisted of 10 μL of staining solution A, 10 μL of staining solution B, 930 μL of staining solution C, and 50 μL of X-Gal. Finally, the cells were washed twice with PBS and suspended in 2 mL of PBS. Three visual fields were randomly selected from each well under a 100 × inverted microscope. The senescence rate = (blue cell number/total cell number) × 100%. The experiment was repeated thrice.

### Flow cytometry analysis of apoptosis

Prepared cells were washed twice with PBS, centrifuged, and resuspended in 200 μL of binding buffer. The cells were incubated with a mixed staining solution (10 μL of Annexin V-FITC and 5 μL of PI, TransGen Biotech, Beijing, China) at room temperature in the dark for 15 min and mixed with 300 μL of binding buffer. A flow cytometer (B&D company) was used to detect apoptosis at an excitation wavelength of 488 nm. The experiment was repeated thrice.

### Caspase-3 activity detection

The activity of caspase-3 was detected using a caspase-3 colorimetric assay kit (AmyJet Scientific Inc.). Caspase-3 catalyzed the substrate Ac-DEVD-pNA to produce yellow pNA (p-nitroaniline). The activity of caspase-3 was expressed as the absorbance of pNA at 400 ~ 410 nm.

### Reactive oxygen species (ROS) detection

Cells growing in a 6-well plate were cultured with 10 μmol/L DCFH-DA probes (1 ml per well; ROS detection kit, Beyotime) at 37 °C in the dark for 20 min and washed 3 times with serum-free medium. Qualitative observation and quantitative detection were performed with an inverted fluorescence microscope and a flow cytometer, respectively. The excitation and emission wavelengths of the DCFH-DA probe were 488 nm and 525 nm respectively.

### ELISA

ELISA kits (R&D, USA) were used for detecting adiponectin, ICAM-1, MCP-1 and IL-1β. Samples to be tested were added into a 96-well ELISA plate and incubated overnight at room temperature. The culture medium was discarded and the plate was washed 3 times with PBS for 5 min each. The samples were incubated with 5% BSA (100 μL/well) for 1 h, primary antibodies (100 μL/well) for 3 h, and HRP-labeled secondary antibodies (100 μL/well) for 1 h. The antibodies were diluted with PBS containing 5% BSA. The samples were washed 3 times with PBS for 5 min each and incubated with 10 μL of substrate at 37 °C for 10 ~ 15 min. The absorbance at 450 nm of each sample was measured. The experiment was repeated thrice.

### Dual-luciferase reporter assay

The binding sites between miR-711 and TLR4 were predicted by the TargetScan database (http://www.targetscan.org/vert_71/). Wild or mutant sequence of the binding site (wt-TLR4 or mut-TLR4) was synthesized and inserted into pGL3-Basic vector which was later delivered together with miR-711 mimic or miR-711 inhibitor into H9C2 cells. The cells were incubated for 48 h and subjected to detection of Firefly luciferase activity and Renilla luciferase activity (phRL-TK vector, as an internal control). The experiment was repeated thrice.

### T2DM rat models

Fifteen healthy specific pathogen-free male Sprague–Dawley rats (2 months old, 190.5 ± 10.7 g) were provided by the Shanghai Laboratory Animal Center of the Chinese Academy of Sciences. The animals ate and drank freely in separate cages. The animal room was provided with natural light and kept at 23 ± 2 °C. This study abided by the rules of animal experiments and obtained approval from the ethics committee of the 3rd Xiangya Hospital of Central South University.

After being fed for 1 week, the rats were weighed and randomly divided into control (C) group (3 rats) and model group (12 rats). The control group was fed a standard diet, while the model group was given high-glucose and high-fat feed. Three weeks later, the rats fasted for 12 h. The tail was cut and blood was taken to measure the glucose level. After the glucose test, the model group was intraperitoneally injected with 60 mg/kg streptozotocin (STZ) (dissolved in citric acid-sodium citrate buffer, 10 g/L), while the control group was injected with the same dose of citrate acid-sodium citrate buffer. Blood glucose tests were performed at the second and fourth weeks. A glucose level of ≥ 16.7 mmol/L indicated successful induction of diabetes. The rats were observed to see if they have polyphagia, polydipsia, polyuria, weight loss, and other symptoms of T2DM. Three rats in the model group failed to meet the standards of a T2DM model. Next, the model rats were randomly divided into diabetes (D) group, diabetes + adiponectin (DA) group, and diabetes + miR-711 inhibitor + adiponectin (DMA) group (3 rats per group). The DA group was intraperitoneally injected with 6 mg/kg adiponectin and the D group was injected with the same dose of normal saline. The DMA group was injected with 1 nmol miR-711 knockdown lentiviruses through tail vein apart from intraperitoneal injection of 6 mg/kg adiponectin. After continuous 7 days of treatment, the rats were subjected to measurement of left ventricular systolic pressure (LVSP), left ventricular end-diastolic pressure (LVEDP), the maximum rate of increase in left ventricular pressure (+ dp/dtmax), and the maximum rate of decrease in left ventricular pressure (−dp/dtmax). The rats were sacrificed one month later.

### Histological exanimation

After blood collection, the rats were sacrificed and their hearts were immediately taken out. The myocardium was cut into 2 ~ 3 mm slices. Slices of the same part of the myocardium were fixed in 10% formaldehyde, soaked in 0.01 mol/L PBS overnight, dehydrated in gradient alcohol, and embedded in paraffin (4 mm). After deparaffinization with xylene and rehydration with gradient ethanol, the slices were stained with hematoxylin and eosin and dehydrated with gradient ethanol and xylene. The slices were mounted with neutral balsam and observed under a microscope.

### TUNEL detection of apoptosis

Paraffin slices were dewaxed with xylene and rehydrated in gradient alcohol (from 95%, 90%, 85%, 75% to 50%). The slices were placed in PBS for 5 min and treated with 100 μL of permeabilization solution at room temperature for 5 min. Labeling solution (50 μL) and TdT (2 μL) were mixed in a 0.5 mL centrifuge tube, added onto the surface of the slices, and reacted in the dark at 37 °C for 1 h. The slices were treated with 100 μL of permeabilization solution at room temperature for 3 × 5 min. Excess liquid was removed and the slices were mounted with 2.5 μL of anti-quenching reagent and observed under a fluorescence microscope.

### Statistical analysis

All data were analyzed by SPSS 25.0 software and expressed as mean ± standard deviation. Group differences were compared by one-way analysis of variance, followed by Tukey’s multiple comparisons test. Differences were regarded as statistically significant if *P* < 0.05. GraphPad 9.0 software was used for making statistical graphs.

## Results

### Adiponectin and miR-711 were underexpressed in high glucose-treated H9c2 cells

**Fig. 1:**
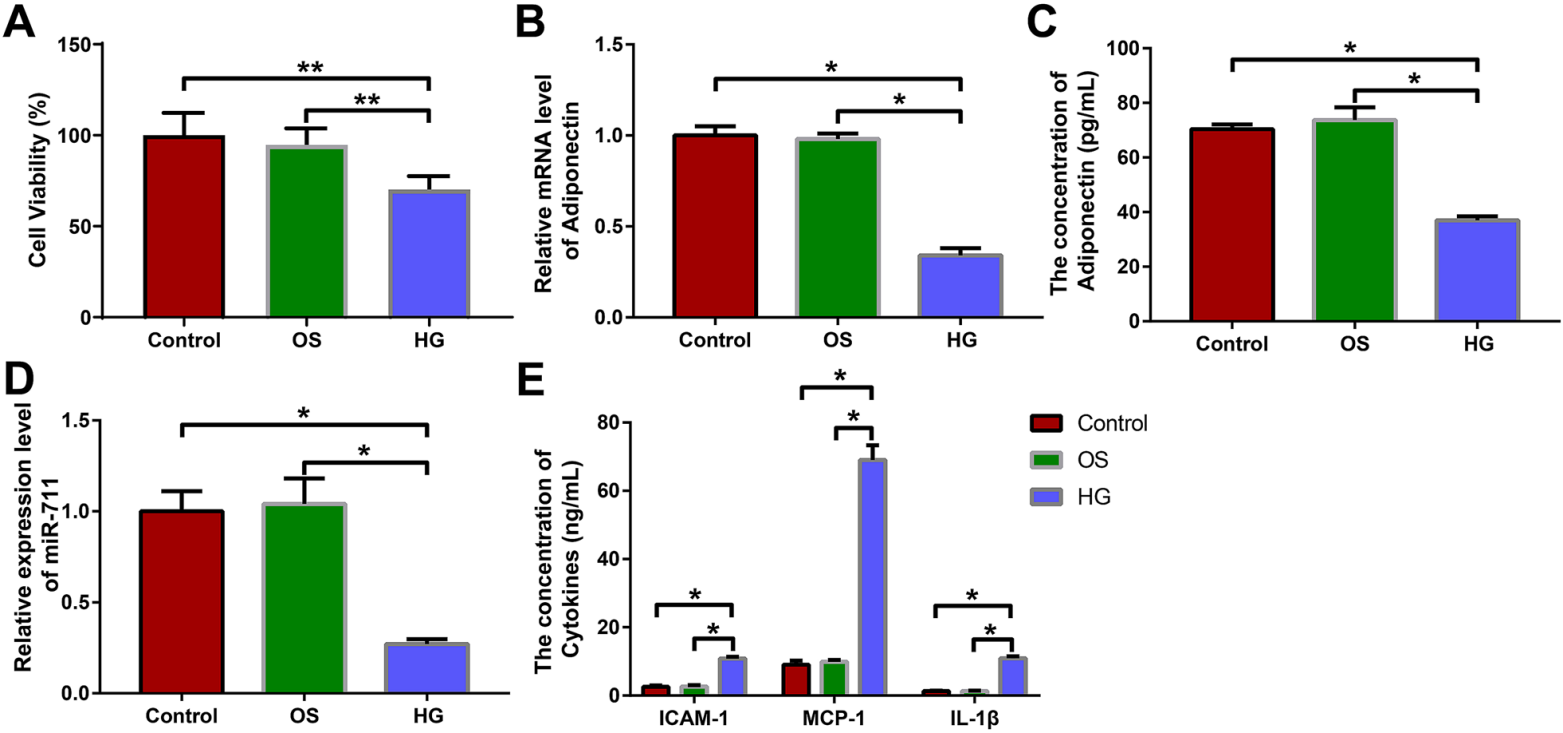
Adiponectin and miR-711 were underexpressed in high glucose-treated H9c2 cells. H9c2 cells were treated with mannitol or high glucose. **A** MTT was used to detect cell viability. **B** qRT-PCR was used to detect the expression of adiponectin in cells. **C** ELISA was performed to detect the expression of adiponectin in cell culture supernatant. **D** qRT-PCR was used to detect the expression of miR-711 in cells. **E** ELISA was performed to detect the expression of ICAM-1, MCP-1, and IL-1β in cell culture supernatant. The data were all measurement data and expressed as mean ± standard deviation. One-way analysis of variance was used for comparisons between multiple groups and Tukey's test for post-hoc multiple comparisons. * indicates *P* < 0.05. The experiments were repeated thrice. *OS* osmotic, *HG* high glucose, MTT, 3-(4,5-dimethylthiazol-2-yl)-2,5-diphenyltetrazolium bromide, *qRT-PCR* quantitative real-time polymerase chain reaction, *ELISA* enzyme-linked immunosorbent assay, *ICAM* intercellular adhesion molecule, *MCP* monocyte chemotactic protein, *IL* interleukin

First we detected the viability of H9c2 cells in the control, OS and HG groups using the MTT method. After 72 h of high glucose treatment, the HG group showed lower viability than the control and OS groups (Fig. 1A, P < 0.05), which indicated that the cells in the HG group were successfully injured by high glucose. Next, we analyzed the expression of adiponectin mRNA and miR-711 in the cells using qRT-PCR and also measured the concentration of adiponectin in the cell culture supernatant using ELISA. Both adiponectin (Fig. 1B-C, P < 0.05) and miR-711 (Fig. 1D, P < 0.05) were lowly expressed in the HG group, compared with those in the control and OS groups. Studies have shown that sustained high glucose treatment can induce cardiomyocyte apoptosis and fibrosis through various cellular cytokines and inflammatory factors, thereby accelerating the progression of diabetes [20–22]. In this context, we used ELISA to detect the adhesion molecule ICAM-1 and pro-inflammatory cytokines MCP-1 and IL-1β in the cell culture supernatant. The levels of ICAM-1, MCP-1 and IL-1β in the HG group were significantly higher than those in the control and OS groups (Fig. 1E, P < 0.05). Furthermore, there were no significant differences in the above experiment results between the control group and OS group (Fig. 1A-E, P > 0.05). Taken together, these results indicate that high glucose treatment impairs the viability of H9c2 cells, decreases the expression of adiponectin and miR-711, and promotes the production of ICAM-1, MCP-1, and IL-1β.

### High glucose treatment impacts on the biological behaviors of H9c2 cells

**Fig. 2:**
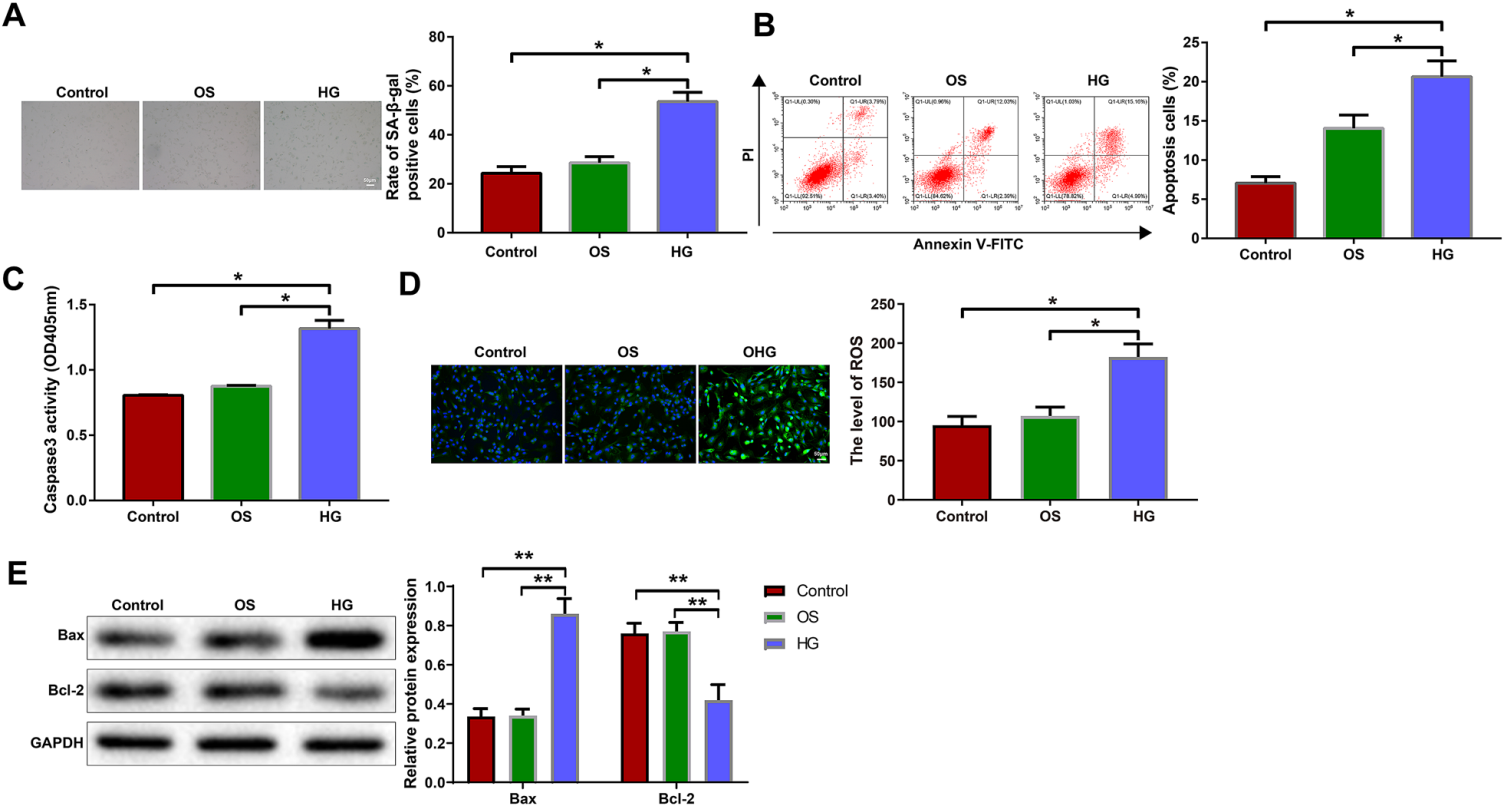
High glucose treatment impacts on the biological behaviors of H9c2 cells. H9c2 cells were treated with mannitol or high glucose. **A** β-galactosidase staining was used to reveal cell senescence. **B** Flow cytometry was used to analyze cell apoptosis. **C** The colorimetric method was used to detect the activity of caspase-3 in cells. **D** DCFH-DA probes were used to detect ROS in cells. **E** Western blotting was used to detect Bax and Bcl-2 in cells. The data were all measurement data and expressed as mean ± standard deviation. One-way analysis of variance was used for comparisons between multiple groups and Tukey's test for post-hoc multiple comparisons. ^*^ indicates *P* < 0.05. The experiments were repeated thrice. *OS* osmotic, *HG* high glucose, DCFH-DA, 2′,7′-dichlorodihydrofluorescein diacetate, *ROS* reactive oxygen species, *Bax* Bcl-2-associated X protein, *Bcl-2* B-cell lymphoma-2

We further analyzed high glucose-induced changes in the biological behaviors of H9c2 cells. β-galactosidase staining and flow cytometry were utilized to detect the senescence and apoptosis of H9c2 cells, respectively. The senescence and apoptosis rates of the HG group were higher than those of the control and OS groups (Fig. 2A, B, P < 0.05). The colorimetric method was used to detect the activity of caspase-3 in the cells. The HG group showed higher caspase-3 activity than the control and OS groups (Fig. 2C, P < 0.05). DCFH-DA probes were used to detect ROS in the cells. The HG group had a higher ROS level than the control and OS groups (Fig. 2D, P < 0.05). Furthermore, as shown by the protein bands, the HG group had higher expression of the pro-apoptotic protein Bax and lower expression of the anti-apoptotic protein Bcl-2 than the control and OS groups (Fig. 2E, P < 0.05). Still, there were no significant differences in the above experiment results between the control group and OS group (Fig. 2A, E, P > 0.05). Collectively, these results indicate that high glucose treatment promotes the senescence and apoptosis of H9c2 cells.

### miR-711 negatively regulates TLR4 and reduces high glucose-provoked inflammatory responses of H9c2 cells

**Fig. 3:**
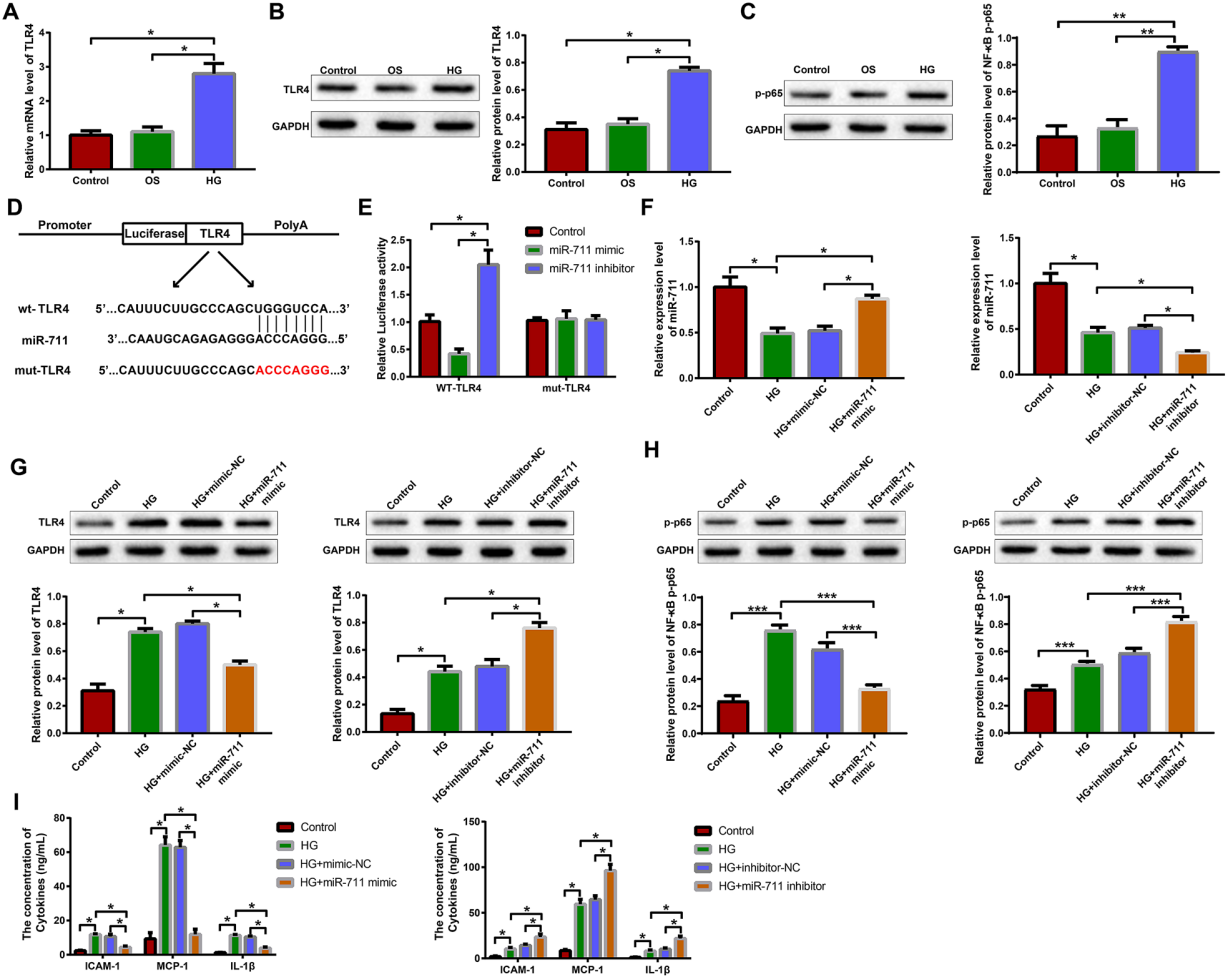
miR-711 negatively regulates TLR4 and reduces high glucose-provoked inflammatory responses of H9c2 cells. **A** qRT-PCR was used to detect the expression of TLR4 mRNA in H9c2 cells treated with mannitol or high glucose. Western blotting was used to detect the expression of TLR4 protein **B** and p-NF-κB p65 protein **C** in H9c2 cells treated with mannitol or high glucose. **D** TargetScan database predicted the binding sites between miR-711 and TLR4 mRNA and mutations were designed. **E** Dual-luciferase reporter assay was performed to verify the binding between miR-711 and TLR4 mRNA. Next, high glucose-treated H9c2 cells were transfected with mimic-NC, miR-711 mimic, inhibitor-NC or miR-711 inhibitor. **F** qRT-PCR was used to detect the expression of miR-711 in the transfected cells. Western blotting was used to detect the expression of TLR4 protein **G** and p-NF-κB p65 protein **H** in the transfected cells. **I** ELISA was performed to detect the concentrations of ICAM-1, MCP-1, and IL-1β in cell culture supernatant. The data were all measurement data and expressed as mean ± standard deviation. One-way analysis of variance was used for comparisons between multiple groups and Tukey’s test for post-hoc multiple comparisons. ^*^ indicates *P* < 0.05. The experiments were repeated thrice. *OS* osmotic, *HG* high glucose, *NC* negative control, *TLR4* toll-like receptor 4, *qRT-PCR* quantitative real-time polymerase chain reaction, *ELISA* enzyme-linked immunosorbent assay, *ICAM* intercellular adhesion molecule, *MCP* monocyte chemotactic protein, *IL* interleukin

TLR4 signaling is a predominant driver of metabolic inflammation [23]. Therefore, we first used qRT-PCR and western blotting to detect the expression of TLR4/NF-κB signaling pathway in high glucose-treated H9c2 cells. The HG group showed higher expression of TLR4 mRNA/protein and p-NF-κB p65 protein than the control and OS groups (Fig. 3A, C, P < 0.05). The TargetScan database (http://www.targetscan.org/vert_71/) predicted there were binding sites between miR-711 and TLR4 mRNA. Wt-TLR4 and mut-TLR4 were synthesized and inserted into the luciferase reporter vectors (Fig. 3D). H9c2 cells transfected with wt-TLR4 and miR-711 mimic exhibited lower luciferase activity than control cells, while those transfected with wt-TLR4 and miR-711 inhibitor showed higher luciferase activity than control cells (Fig. 3E, P < 0.05). No significant difference in the luciferase activity was observed in cells transfected with either mut-TLR4 and miR-711 mimic or mut-TLR4 and miR-711 inhibitor (Fig. 3E). The above results indicate that miR-711 can bind TLR4 mRNA and inhibit the expression of TLR4 protein. Next, we introduced mimic-NC, miR-711 mimic, inhibitor-NC or miR-711 inhibitor into high glucose-treated H9c2 cells and successfully elevated or decreased the expression of miR-711 in the cells (Fig. 3F, P < 0.05). The western blot analysis showed that overexpression of miR-711 inhibited the expression of TLR4 and p-NF-κB p65, while inhibition of miR-711 promoted the expression of TLR4 and p-NF-κB p65 (Fig. 3G, H, P < 0.05). The results of ELISA showed that overexpression of miR-711 reduced the production of ICAM-1, MCP-1, and IL-1β by high glucose-treated H9c2 cells, while inhibition of miR-711 exacerbated this phenomenon (Fig. 3I, P < 0.05). Taken together, these results indicate that overexpression of miR-711 can block TLR4/NF-κB pathway and attenuate inflammatory signals in high glucose-treated H9c2 cells.

### miR-711 affects the biological behaviors of high glucose-treated H9c2 cells by regulating TLR4/NF-κB

**Fig. 4:**
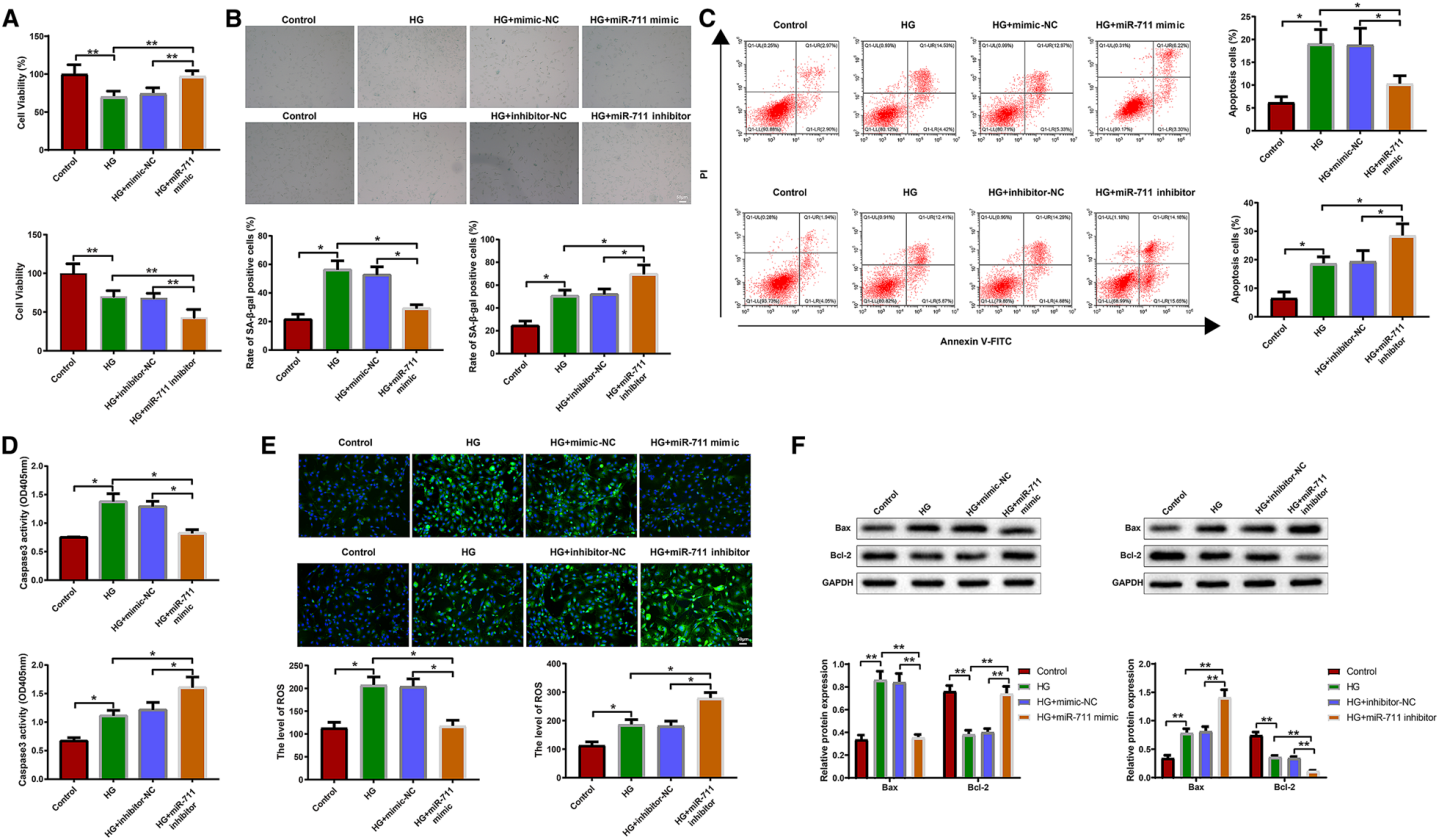
miR-711 affects the biological behaviors of high glucose-treated H9c2 cells by regulating TLR4/NF-κB. High glucose-treated H9c2 cells were transfected with mimic-NC, miR-711 mimic, inhibitor-NC or miR-711 inhibitor. **A** MTT assay was performed to assess cell viability. **B** β-galactosidase staining was used to reveal cell senescence. **C** Flow cytometry was used to analyze cell apoptosis. **D** The colorimetric method was used to detect the activity of caspase-3 in cells. **E** DCFH-DA probes were used to detect ROS in cells. **F** Western blotting was used to detect Bax and Bcl-2 in cells. The data were all measurement data and expressed as mean ± standard deviation. One-way analysis of variance was used for comparisons between multiple groups and Tukey's test for post-hoc multiple comparisons. ^*^ indicates *P* < 0.05. The experiments were repeated thrice. *HG* high glucose, *NC* negative control, *TLR4* toll-like receptor 4, *NF-κB* nuclear factor κB, MTT, 3-(4,5-dimethylthiazol-2-yl)-2,5-diphenyltetrazolium bromide; DCFH-DA, 2′,7′-dichlorodihydrofluorescein diacetate; *ROS* reactive oxygen species, *Bax* Bcl-2-associated X protein; *Bcl-2* B-cell lymphoma-2

To determine the effects of miR-711 on the biological behaviors of high glucose-treated H9c2 cells, we used MTT, β-galactosidase staining, flow cytometry, colorimetric method, DCFH-DA probes, and western blotting to analyze viability, senescence, apoptosis, and related protein expression in high glucose-treated H9c2 cells transfected with mimic-NC, miR-711 mimic, inhibitor-NC or miR-711 inhibitor. Overexpression of miR-711 increased the viability (Fig. 4A, P < 0.05) and slowed the senescence and apoptosis (Fig. 4B, C, P < 0.05) of high glucose-treated H9c2 cells. Moreover, overexpression of miR-711 reduced caspase-3 activity (Fig. 4D, P < 0.05), ROS level (Fig. 4E, P < 0.05), and Bax expression (Fig. 4F, P < 0.05) in high glucose-treated H9c2 cells. The expression trend of Bcl-2 was opposite to that of Bax (Fig. 4F, P < 0.05). Inhibition of miR-711 exacerbated high glucose-induced impairment of the biological behaviors of H9c2 cells (Fig. 4A, F, P < 0.05). From the current and last results, miR-711 might increase viability and suppress senescence and apoptosis in high glucose-treated H9c2 cells through blockade of TLR4/NF-κB pathway.

### Adiponectin regulates TLR4 inflammatory signaling through miR-711

**Fig. 5:**
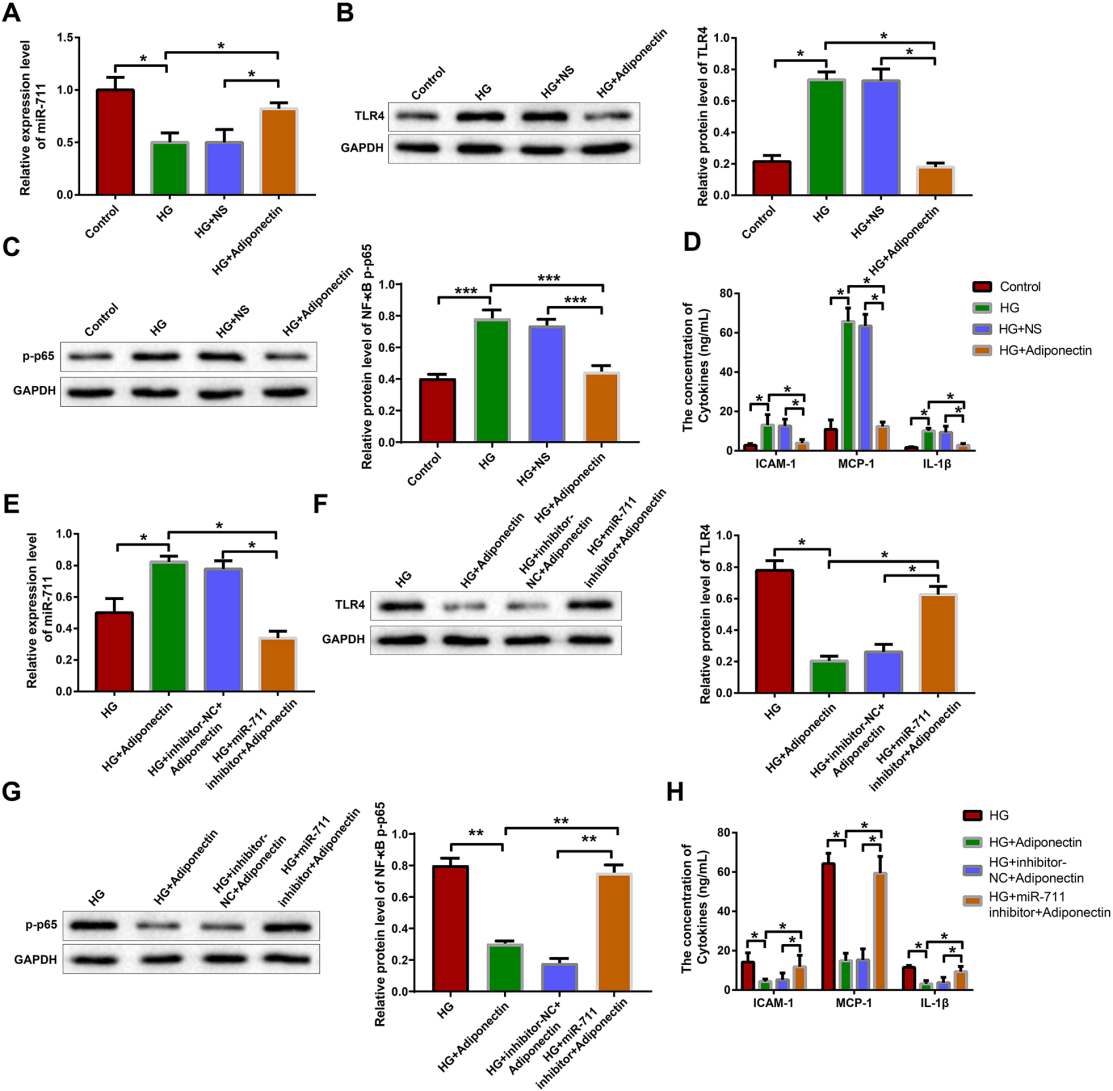
Adiponectin regulates TLR4 inflammatory signaling through miR-711. H9c2 cells cultured in high glucose medium were treated with NS or adiponectin: **A** qRT-PCR was used to detect the expression of miR-711. Western blotting was used to detect the expression of TLR4 protein **B** and p-NF-κB p65 protein **(C)**. **D** ELISA was performed to detect the levels of ICAM-1, MCP-1, and IL-1β in cell culture supernatant. Diabetic H9c2 cells were treated adiponectin and transfected with miR-711 inhibitor or inhibitor-NC: **E** qRT-PCR was used to detect the expression of miR-711. Western blotting was used to detect the expression of TLR4 protein **F** and p-NF-κB p65 protein **(G)**. **H** ELISA was performed to detect the levels of ICAM-1, MCP-1, and IL-1β in cell culture supernatant. The data were all measurement data and expressed as mean ± standard deviation. One-way analysis of variance was used for comparisons between multiple groups and Tukey’s test for post-hoc multiple comparisons. ^*^ indicates *P* < 0.05. The experiments were repeated thrice. *HG* high glucose, *NS* normal saline, *NC* negative control, *TLR4* toll-like receptor 4, *qRT-PCR* quantitative real-time polymerase chain reaction, *ELISA* enzyme-linked immunosorbent assay, *ICAM* intercellular adhesion molecule, *MCP* monocyte chemotactic protein, *IL* interleukin

First we explored the function of adiponectin in diabetic cardiomyocytes. H9c2 cells cultured in high glucose medium were treated with normal saline or adiponectin. Adiponectin treatment increased the expression of miR-711 (Fig. 5A, P < 0.05) and decreased that of TLR4 and p-NF-κB p65 proteins (Fig. 5B, C, P < 0.05). The results of ELISA showed that adiponectin treatment reduced the levels of ICAM-1, MCP-1, and IL-1β in the culture supernatant of high glucose-treated H9c2 cells (Fig. 5D, P < 0.05).

Next, we delivered miR-711 inhibitor or inhibitor-NC into adiponectin-treated diabetic H9c2 cells and divided these cells into HG + miR-711 inhibitor + adiponectin group and HG + inhibitor-NC + adiponectin group. The expression of miR-711 decreased and that of TLR4 and p-NF-κB p65 proteins increased in the HG + miR-711 inhibitor + adiponectin group, compared with those in the HG + inhibitor-NC + adiponectin group (Fig. 5E, G, P < 0.05). The levels of ICAM-1, MCP-1, and IL-1β were elevated in the HG + miR-711 inhibitor + adiponectin group, compared with those in the HG + inhibitor-NC + adiponectin group (Fig. 5H, P < 0.05). Inhibition of miR-711 counteracted the effect of adiponectin treatment on high glucose-induced inflammatory response of H9c2 cells. In another words, adiponectin worked upstream miR-711 and inhibited high glucose-induced inflammatory response of H9c2 cells through miR-711/TLR4/NF-κB.

### Adiponectin/miR-711 regulates myocardial inflammation and apoptosis in diabetic rats

**Fig. 6:**
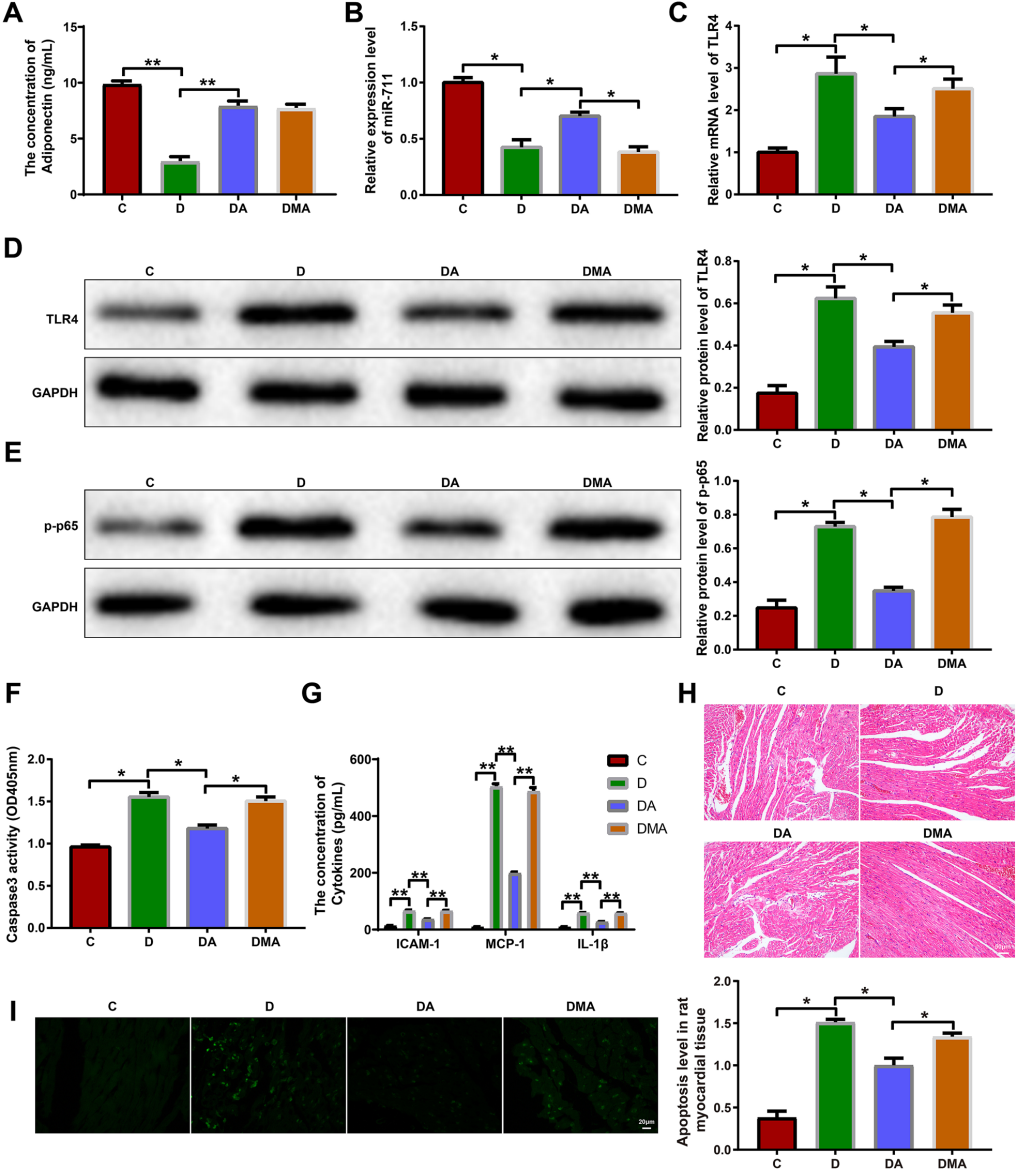
Adiponectin/miR-711 regulates myocardial inflammation and apoptosis in diabetic rats. Diabetic rats were injected with adiponectin or miR-711 + adiponectin. **A** ELISA was used to determine the contents of serum adiponectin. Quantitative real-time polymerase chain reaction was used to detect the expression of miR-711 **B** and TLR4 mRNA **C** in the blood of the rats. Western blotting was used to detect TLR4 **D** and p-NF-κB p65 **E** in the myocardium of the rats. **F** The colorimetric method was used to detect the activity of caspase-3 in the myocardium of the rats. **G** ELISA was performed to detect the concentrations of serum ICAM-1, MCP-1, and IL-1β. **H** Hematoxylin and eosin staining was used to reveal myocardial structure. **I** TUNEL assay was used to reveal myocardial apoptosis. n = 3. The data were all measurement data and expressed as mean ± standard deviation. One-way analysis of variance was used for comparisons between multiple groups and Tukey’s test for post-hoc multiple comparisons. ^*^ indicates *P* < 0.05. *C* control group, *D* diabetes group, *DA* diabetes + adiponectin group, *DMA* diabetes + miR-711 inhibitor + adiponectin group, *TLR4* toll-like receptor 4, *ICAM* intercellular adhesion molecule, *MCP* monocyte chemotactic protein, *IL* interleukin, *TUNEL* terminal deoxynucleotidyl transferase-mediated dUTP-biotin nick end labeling

Finally, we explored the function of the adiponectin/miR-711 axis in animals. The rats were divided into 4 groups: control (C), diabetes (D), diabetes + adiponectin (DA), and diabetes + miR-711 inhibitor + adiponectin (DMA) groups. The D group showed a significantly higher blood glucose level than the C group (Table 2), which indicated successful establishment of a T2DM rat model. The hemodynamic assessment showed that the D group had higher LVEDP and lower LVSP, + dp/dtmax, and -dp/dtmax than the C group (Table 3). However, compared with the D group, the DA or DMA group exhibited no significant difference in the hemodynamic parameters (Table 3), which indicated that adiponectin treatment did not impact on the hemodynamics of diabetic rats. The ELISA analysis showed that the content of serum adiponectin decreased in the D group (vs the C group) and increased in the DA and DMA groups (vs the D group); the difference between the DA group and DMA group was not significant (Fig. 6A, P < 0.05). The qRT-PCR analysis showed that compared with the C group, the D group had lower miR-711 expression and higher TLR4 mRNA expression; these expression trends were reversed by adiponectin treatment in the DA group. Compared with the DA group, the DMA group showed lower miR-711 expression and higher TLR4 expression (Fig. 6B, C, P < 0.05). Western blotting was used to detect the expression of TLR4 and p-NF-κB p65 proteins in the myocardium of these rats. TLR4 and p-NF-κB p65 were upregulated in the D group (vs the C group) and DMA group (vs the DA group) and downregulated in the DA group (vs the D group) (Fig. 6D, E, P < 0.05). The colorimetric method was adopted to measure the activity of caspase-3 in the myocardium. The activity of caspase-3 increased in the D group (vs the C group) and decreased in the DA group (vs the D group); however, the DMA group showed a higher caspase-3 activity than the DA group (Fig. 6F, P < 0.05). As shown by the ELISA assay, the serum levels of ICAM-1, MCP-1, and IL-1β were elevated in the D group (vs the C group) and DMA group (vs the DA group) and reduced in the DA group (vs the D group) (Fig. 6G, P < 0.05). The histological images showed that the C group had tightly and neatly arranged myocardial cells, in which nuclei were stained and clearly visible (Fig. 6H). There were disarranged, hypertrophic and deformed myocardial cells with deeply stained nuclei, fibrotic interstitum, and inflammatory cell infiltration in the myocardium of the D and DMA groups; the histological disorders were partially alleviated in the DA group (Fig. 6H). The TUNEL method was used to detect apoptosis in the myocardium. The rate of myocardial apoptosis rose in the D group (vs the C group) and DMA group (vs the DA group) and declined in the DA group (vs the D group) (Fig. 6I, P < 0.05). Collectively, these data indicate that adiponectin reduced myocardial inflammation and apoptosis in diabetic rats by regulating the miR-711/TLR4/NF-κB signaling pathway.

**Table 2:** Blood glucose test

	C	D	DA	DMA
Glucose concentration (mmol/L)	6.20 ± 0.51	22.05 ± 5.17 ^**a**^	23.78 ± 2.72 ^**a**^	31.17 ± 1.06 ^**a**^

*C* control, *D* diabetes, *DA* diabetes + adiponectin, *DMA* diabetes + miR-711 inhibitor + adiponectin

^**a**^*P* < 0.05, compared with the C group

**Table 3:** Hemodynamics assessment (mmHg)

	LVSP	+ dp/dt	−dp/dt	LVEDP
C	126.03 ± 4.56	603.85 ± 6.59	623.03 ± 8.59	10.03 ± 0.91
D	103.26 ± 4.08 ^**a**^	513.86 ± 10.06 ^**a**^	430.48 ± 27.37 ^**a**^	17.65 ± 1.74 ^**a**^
DA	103.96 ± 3.85 ^**a**^	514.01 ± 8.86 ^**a**^	430.98 ± 25.58 ^**a**^	16.98 ± 1.55 ^**a**^
DMA	102.83 ± 5.02 ^**a**^	510.43 ± 9.45 ^**a**^	396.78 ± 28.12 ^**a**^	17.02 ± 1.02 ^**a**^

*C* control, *D* diabetes, *DA* diabetes + adiponectin, *DMA* diabetes + miR-711 inhibitor + adiponectin, *LVSP* left ventricular systolic pressure, + dp/dt, the maximum rate of increase in left ventricular pressure, −dp/dt, the maximum rate of decrease in left ventricular pressure, *LVEDP* left ventricular end-diastolic pressure

^**a**^*P* < 0.05, compared with the C group

## Discussion

Exposure to hyperglycemia can reduce cardiomyocyte function and contribute to heart failure [24]. Although the current therapies for diabetic cardiomyopathy focus on the control of glucose homeostasis, considerable attention has been attached to dysregulated molecules that can be targeted to induce therapeutic effects [25]. This study established diabetes models using rats and H9c2 cells in which the function of adiponectin/miR-711/TLR4 axis was analyzed. Through bioinformatics analysis and in vitro experiments, miR-711 was identified as a direct and negative regulator of TLR4. Adiponectin was found to work upstream miR-711 and regulate cardiomyocyte behaviors through miR-711/TLR4.

MiRNAs have shown significant regulatory effects on cardiac hypertrophy, cardiomyocyte apoptosis, myocardial fibrosis, oxidative stress, and other pathophysiological processes in diabetic cardiomyopathy [26]. miR-711 is a versatile molecule that mediates neuronal apoptosis, neurodegenerative pathways, tumor cell behaviors, and liver injury [27–32]. Studies have also reported the role of miR-711 in regulating cardiomyocyte apoptosis. For example, miR-711 promoted oxidative stress-induced cardiomyocyte apoptosis by regulating Ang-1, FGF14, and Cacna1c genes [33]. miR-711 was upregulated by PPARγ post myocardial infarction and promoted endoplasmic reticulum stress-induced cardiomyocyte apoptosis by targeting calnexin [34]. Inhibition of miR-711 reduced NF-κB expression to alleviate cardiomyocyte apoptosis during ischemia–reperfusion injury [35]. In contrast, this study found miR-711 was downregulated in high glucose-treated cardiomyocytes. Overexpression of miR-711 enhanced viability and suppressed inflammatory response, senescence, and apoptosis of high glucose-treated cardiomyocytes. More importantly, miR-711 could bind TLR4 mRNA and inhibit the expression of TLR4 and its downstream NF-κB.

TLR4 is a representative TLR family member that can recognize both pathogen- and damage-associated molecular patterns and subsequently trigger rapid inflammatory reactions [36]. TLR is an upstream immunoreceptor in the canonical pathway of NF-κB activation which depends on the phosphorylation and degradation of IκBα and subsequent nuclear translocation of p65:p50 dimers [37]. In this study, TLR4/NF-κB signaling pathway was activated in cardiomyocytes under high glucose conditions. TLR4 can be activated not only by pathogen components (e.g. LPS) but also by mammalian endogenous molecules such as heat-shock proteins (HSPs) [38, 39]. It has been shown that high glucose can induce HSPs [40, 41]. Based on these findings, hyperglycemia may activate TLR4 through endogenous HSPs, thereby stimulating the inflammatory response of cardiomyocytes. TLR4/NF-κB signaling pathway plays an important role in cardiomyocyte inflammation and apoptosis and can be targeted to alleviate myocardial ischemia/reperfusion injury and diabetic cardiomyopathy [42–47]. Therefore, miR-711 might control the inflammatory response and biological behaviors of high glucose-treated cardiomyocytes by blocking TLR4/NF-κB signaling pathway. Apart from miR-711, adiponectin was also found to be downregulated in high glucose-treated cardiomyocytes.

In human body, adiponectin circulates in blood and exerts insulin-sensitizing, anti-atherogenic, and anti-inflammatory effects [48]. The cardioprotective role of adiponectin has been described by many studies. For example, adiponectin strengthened mitochondrial bioenergetics in cardiac myocytes by increasing the activity and assembly of succinate dehydrogenase [49]. Pioglitazone, a PPARγ agonist, enhanced the therapeutic effects of adipose tissue-derived regenerative cells on chronic myocardial infarction by stimulating adiponectin paracrine activity and M2 macrophage polarization [50]. An adiponectin agonist reduced doxorubicin-induced oxidative stress and cardiomyocyte apoptosis by regulating Nrf2 and sirtuin 2 [51]. The protective roles of adiponectin are mediated through suppression of inflammasomes in different cell types [52–54]. A study by Boursereau and colleagues showed that adiponectin decreased the expression of NLRP3 inflammasome through miR-711 and attenuated muscle inflammation in Duchenne muscular dystrophy [55]. Activation of TLR4 can induce formation of NLRP3 inflammasome within cells [56]. In this study, adiponectin increased the expression of miR-711 and decreased that of TLR4/NF-κB signaling pathway and pro-inflammatory cytokine IL-1β, suggesting a possible role of inflammasome inactivation in adiponectin-mediated protection of cardiomyocytes. Adiponectin treatment reduced pathophysiological changes and apoptosis in the myocardium of diabetic rats. Inhibition of miR-711 counteracted the effects of adiponectin on myocardial inflammation and apoptosis.

## Conclusion

In summary, adiponectin inhibits high glucose-induced inflammatory response and apoptosis of cardiomyocytes partially by blocking TLR4/NF-κB signaling pathway through miR-711. The findings support the potential of using exogenous adiponectin for treating diabetic cardiomyopathy and also reveal a molecular mechanism for this complication. Future research can validate the effects of adiponectin in clinical settings based on the current findings. Other targets of miR-711 in regulating diabetic cardiomyocyte apoptosis are also worthy of investigations.

## Data Availability

The datasets used or analyzed during the current study are available from the corresponding author on reasonable request.
